# The Effects of Imitation Dynamics on Vaccination Behaviours in SIR-Network Model

**DOI:** 10.3390/ijerph16142477

**Published:** 2019-07-11

**Authors:** Sheryl Le Chang, Mahendra Piraveenan, Mikhail Prokopenko

**Affiliations:** 1 Complex Systems Research Group, Faculty of Engineering, The University of Sydney, Sydney, NSW 2006, Australia; 2 Charles Perkins Centre, The University of Sydney, John Hopkins Drive, Camperdown, NSW 2006, Australia; 3 Marie Bashir Institute for Infectious Diseases and Biosecurity, The University of Sydney, Westmead, NSW 2145, Australia

**Keywords:** vaccination, epidemic modelling, SIR model, strategy imitation, herd immunity, Erdös-Rényi random networks, greater Sydney commuting network

## Abstract

We present a series of SIR-network models, extended with a game-theoretic treatment of imitation dynamics which result from regular population mobility across residential and work areas and the ensuing interactions. Each considered SIR-network model captures a class of vaccination behaviours influenced by epidemic characteristics, interaction topology, and imitation dynamics. Our focus is the resultant vaccination coverage, produced under voluntary vaccination schemes, in response to these varying factors. Using the next generation matrix method, we analytically derive and compare expressions for the basic reproduction number $R_{0}$ for the proposed SIR-network models. Furthermore, we simulate the epidemic dynamics over time for the considered models, and show that if individuals are sufficiently responsive towards the changes in the disease prevalence, then the more expansive travelling patterns encourage convergence to the endemic, mixed equilibria. On the contrary, if individuals are insensitive to changes in the disease prevalence, we find that they tend to remain unvaccinated. Our results concur with earlier studies in showing that residents from highly connected residential areas are more likely to get vaccinated. We also show that the existence of the individuals committed to receiving vaccination reduces $R_{0}$ and delays the disease prevalence, and thus is essential to containing epidemics.

## 1. Introduction

Vaccination has long been established as a powerful tool in managing and controlling infectious diseases by providing protection to susceptible individuals [1]. With a sufficiently high vaccination coverage, the probability of the remaining unvaccinated individuals getting infected reduces significantly. However, such systematic programs by necessity may limit the freedom of choice of individuals. When vaccination programs are made voluntary, the vaccination uptake declines as a result of individuals choosing not to vaccinate, as seen in Britain in 2003 when the vaccination program for Measles-Mumps-Rubella (MMR) was made voluntary [2]. Parents feared possible complications from vaccination [2,3] and hoped to exploit the ‘*herd immunity*’ by assuming other parents would choose to vaccinate their children. Such hopes did not materialize precisely because other parents also thought similarly.

Under a voluntary vaccination policy an individual’s decision depends on several factors: the social influence from one’s social network, the risk perception of vaccination, and the risk perception of infection, in terms of both likelihood and impact [4]. This decision-making is often modelled using game theory [3,5,6,7,8,9,10,11], by allowing individuals to compare the cost of vaccination and the potential cost of non-vaccination (in terms of the likelihood and impact of infection), and adopting imitation dynamics in modelling the influence of social interactions. However, many of the earlier studies [12,13,14] were based on the key assumption of a well-mixed homogeneous population where each individual is assumed to have an equal chance of making contact with any other individual in the population. This population assumption is rather unrealistic as large populations are often diverse with varying levels of interactions. To address this, more recent studies [7,8,9,10,11,15] model populations as complex networks where each individual, represented by a node, has a finite set of contacts, represented by links [16].

It has been shown that there is a critical cost threshold in the vaccination cost above which the likelihood of vaccination drops steeply [6]. In addition, highly connected individuals were shown to be more likely to choose to be vaccinated as they perceive themselves to be at a greater risk of being infected due to high exposure within the community. In modelling large-scale epidemics, the population size (i.e., number of nodes) can easily reach millions of individuals, interacting in a complex way. The challenge, therefore, is to extend epidemic modelling not only with the individual vaccination decision-making, but also capture diverse interaction patterns encoded within a network. Such an integration has not yet been formalized, motivating our study. Furthermore, once an integrated model is developed, a specific challenge is to consider how the vaccination imitation dynamics developing across a network affects the basic reproduction number, $R_{0}$. This question forms our second main objective.

One relevant approach partially addressing this objective is offered by the multi-suburb (or multi-city) SIR-network model [17,18,19,20,21] where each node represents a neighbourhood (or city) with a certain number of residents. The daily commute of individuals between two neighbourhoods is modelled along the network link connecting the two nodes, which allows to quantify the disease spread between individuals from different neighbourhoods. Ultimately, this model captures meta-population dynamics in a multi-suburb setting affected by an epidemic spread at a greater scale. To date, these models have not yet considered intervention (e.g., vaccination) options, and the corresponding social interaction across the populations.

To incorporate the imitation dynamics, modelled game-theoretically, within a multi-suburb model representing mobility, we propose a series of integrated vaccination-focused SIR-network models. This allows us to systematically analyze how travelling patterns affect the voluntary vaccination uptake due to adoption of different imitation choices, in a large distributed population. The developed models use an increasingly complex set of vaccination strategies. Thus, our specific contribution is the study of the vaccination uptake, driven by imitation dynamics under a voluntary vaccination scheme, using an SIR model on a complex network representing a multi-suburb environment, within which the individuals commute between residential and work areas.

In Section 2.2, we present the models and methods associated with this study: in particular, we present analytical derivations of the basic reproduction number $R_{0}$ for the proposed models using the Next Generation Operator Approach [22], and carry out a comparative analysis of $R_{0}$ across these models. In Section 3, we simulate the epidemic and vaccination dynamics over time using the proposed models in different network settings, including a pilot case of a 3-node network, a 3000-node Erdös-Rényi random network and a real-world commuting network in Greater Sydney area generated from 2016 Australian census data. The comparison of the produced results across different models and settings is carried out for the larger network, with the focus on the emergent attractor dynamics, in terms of the proportion of vaccinated individuals. Particularly, we analyze the sensitivity of the individual strategies (whether to vaccinate or not) to the levels of disease prevalence produced by the different considered models. Section 4 concludes the study with a brief discussion of the importance of these results.

## 2. Materials and Methods

### 2.1. Technical Background

#### 2.1.1. Basic Reproduction Number $R_{0}$

The basic reproduction number $R_{0}$ is defined as the number of secondary infections produced by an infected individual in an otherwise completely susceptible population [23]. It is well-known that $R_{0}$ is an epidemic threshold, with the disease dying out as $R_{0}<1$, or becoming endemic as $R_{0}>1$ [17,24]. This finding strictly holds only in deterministic models with infinite population [25]. The topology of the underlying contact network is known to affect the epidemic threshold [26]. Many disease transmission models have shown important correlations between $R_{0}$ and the key epidemic characteristics (e.g., disease prevalence, attack rates, etc.) [27]. In addition, $R_{0}$ has been considered as a critical threshold for phase transitions studied with methods of statistical physics or information theory [28].

#### 2.1.2. Vaccination Model with Imitation Dynamics

Imitation dynamics, a process by which individuals copy the strategy of other individuals, is widely used to model vaccinating behaviours incorporated with SIR models. The model proposed in [3] applied game theory to represent parents’ decision-making about whether to get their newborns vaccinated against childhood disease (e.g., measles, mumps, rubella, pertussis). In this model, individuals are in a homogeneously mixing population, and susceptible individuals have two ‘pure strategies’ regarding vaccination: to vaccinate or not to vaccinate. The non-vaccination decision can change to the vaccination decision at a particular sampling rate, however the vaccination decision cannot be changed to non-vaccination. Individuals adopt one of these strategies by weighing up their perceived payoffs, measured by the probability of morbidity from vaccination, and the risk of infection respectively. The payoff for vaccination ($f_{v}$) is given as,

(1) $$f_{v}=-r_{v}$$

and the payoff for non-vaccination ($f_{nv}$), measured as the risk of infection, is given as

(2) $$f_{nv}=-r_{nv}mI\left(t\right)$$

where $r_{v}$ is the perceived risk of morbidity from vaccination, $r_{nv}$ is the perceived risk of morbidity from non-vaccination (i.e., infection), I(t) is the current disease prevalence in population fraction at time *t*, and *m* is the sensitivity to disease prevalence [3].

From Equations (1) and (2), it can be seen that the payoff for vaccinated individuals is a simple constant, and the payoff for unvaccinated individuals is proportional to the extent of epidemic prevalence (i.e., the severity of the disease).

It is assumed that life-long immunity is granted with effective vaccination. If one individual decides to vaccinate, s/he cannot revert back to the unvaccinated status. To make an individual switch to a vaccinating strategy, the payoff gain, $\Delta E=f_{v}-f_{nv}$ must be positive (ΔE>0). Let *x* denote the relative proportion of vaccinated individuals, and assume that an unvaccinated individual can sample a strategy from a vaccinated individual at certain rate σ, and can switch to the ‘vaccinate strategy’ with probability ρΔE. Then the time evolution of *x* can be defined as:

(3) $$\begin{array}{cc} \dot{x} & =(1-x)\sigma x\rho \Delta E\\ & =\delta x\left(1-x\right)\left[-r_{v}+r_{nv}mI\right] \end{array}$$

where δ=σρ can be interpreted as the combined imitation rate that individuals use to sample and imitate strategies of other individuals. Equation (3) can be rewritten so that *x* only depends on two parameters:

(4) $$\dot{x}=\kappa x\left(1-x\right)\left(-1+\omega I\right)$$

where $\kappa =\delta r_{v}$ and $\omega =mr_{nv}/r_{v}$, assuming that the risk of morbidity from vaccination, $r_{v}$, and the risk of morbidity from non-vaccination (i.e., morbidity from infection), $r_{nv}$, are constant during the course of epidemics. Hence, κ is the adjusted imitation rate, and ω measures individual’s responsiveness towards the changes in disease prevalence.

The disease prevalence *I* is determined from a simple SIR model [23] with birth and death that divides the population into three health compartments: *S* (susceptible), *I* (infected) and *R* (recovered). If a susceptible individual encounters an infected individual, s/he contracts the infection with transmission rate β, and thus progresses to the infected compartment, while an infected individual recovers at rate γ. It is assumed that the birth and death rate are equal, denoted by μ [29]. Individuals in all compartments die at an equal rate, and all newborns are added to the susceptible compartment unless and until they are vaccinated. Vaccinated newborns are moved to the recovered class directly, with a rate μx. Therefore, the dynamics are modelled as follows [3]:

(5) $$\begin{array}{cc} \dot{S} & =\mu (1-x)-\beta SI-\mu S\\ \dot{I} & =\beta SI-\gamma I-\mu I\\ \dot{R} & =\mu x+\gamma I-\mu R\\ \dot{x} & =\kappa x(1-x)(-1+\omega I) \end{array}$$

where *S*, *I*, and *R* represent the proportion of susceptible, infected, and recovered individuals respectively (that is, S+I+R=1), and *x* represents the proportion of vaccinated individuals within the susceptible class at a given time. The variables *S*, *I*, and *R*, as well as *x*, are time-dependent, but for simplicity, we omit subscript *t* for these and other time-dependent variables.

Model (5) predicts oscillations in vaccine uptake in response to changes in disease prevalence. It is found that oscillations are more likely to occur when individuals imitate each other more quickly (i.e., higher κ). The oscillations are observed to be more volatile when people alter their vaccinating behaviours promptly in response to changes in disease prevalence (i.e., higher ω). Overall, higher κ or ω produce stable limit cycles at greater amplitude. Conversely, when individuals are insensitive to changes in disease prevalence (i.e., low ω), or imitate at a slower rate (i.e., low κ), the resultant vaccinating dynamics converge to equilibrium [3].

#### 2.1.3. Multi-City Epidemic Model

**Figure 1:**
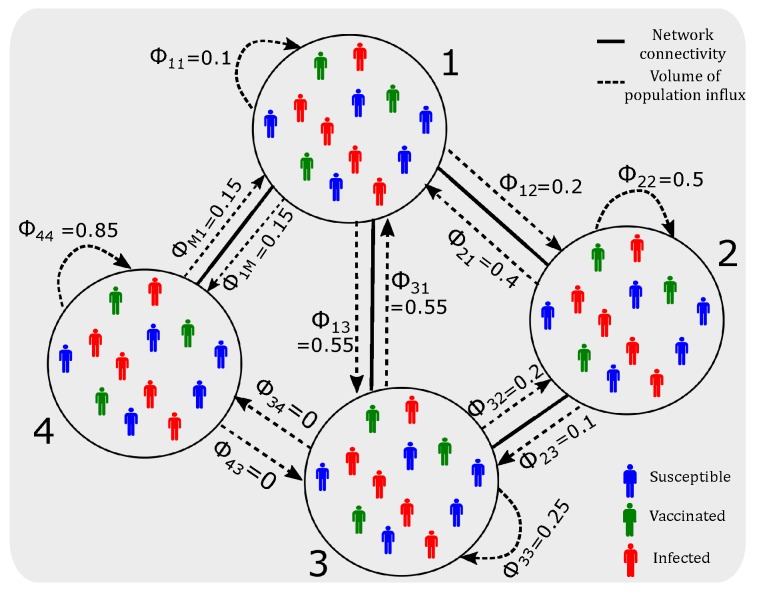
Schematic of daily population travel dynamics across different suburbs (nodes): a 4-node example. Solid line: network connectivity. Dashed line: volume of population flux (influx and outflux). Non-connected nodes have zero population flux (e.g., $\phi_{34}=\phi_{43}=0$). For each node, the daily outflux proportions (including travel to the considered node itself) sum up to unity, however, the daily influx proportions do not.

Several multi-city epidemic models [17,18,19] consider a network of suburbs as *M* nodes, in which each node i∈V represents a suburb, where i=1,2,⋯M. If two nodes i,j∈V are linked, a fraction of population living in node *i* can travel to node *j* and back (commute from *i* to *j*, for example for work) on a daily basis. The connectivity of suburbs and the fraction of people commuting between them are represented by the population flux matrix ϕ whose entries represent the fraction of population daily commuting from *i* to *j*, $\phi_{ij}\in \left[0,1\right]$ (Equation (6)). Note that $\phi_{ij}\neq \phi_{ji}$, thus ϕ is not a symmetric matrix. Figure 1 shows an example of such travel dynamics.

(6) $$\phi_{M\times M}=\left[\begin{array}{cccc} \phi_{11} & \phi_{12} & \cdots & \phi_{1M}\\ \phi_{21} & \phi_{22} & \cdots & \phi_{2M}\\ \vdots & \vdots & \ddots & \vdots \\ \phi_{M1} & \phi_{N2} & \cdots & \phi_{MM} \end{array}\right]$$

Trivially, each row in ϕ, representing proportions of the population of a suburb commuting to various destinations, has to sum up to unity:

(7) $$\sum_{j=1}^{M}\phi_{ij}=1\;,\;\;\forall i\in V$$

However, the column sum measures the population influx to a suburb during a day, and therefore depends on the node’s connectivity. Column summation in ϕ does not necessarily equal to unity.

It is also necessary to differentiate between *present population* $N_{j}^{p}$ and *native population* $N_{j}$ in node *j* on a particular day. $N_{j}^{p}$ is used as a normalizing factor to account for the differences in population flux for different nodes:

(8) $$N_{j}^{p}=\sum_{k=1}^{M}\phi_{kj}N_{k}$$

Assuming the disease transmission parameters are identical in all cities, the standard incidence can be expressed as [17]:

(9) $$\sum_{j=1}^{M}\sum_{k=1}^{M}\lambda_{j}\beta_{j}\frac{I_{k}}{N_{j}^{p}}S_{i}$$

where $I_{l}$ and $S_{l}$ denote respectively the number of infective and susceptible individuals in city *l*, and $\lambda_{l}$ is the average number of contacts in city *l* per unit time. Incorporating travelling pattern defined by ϕ, yields [19]:

(10) $$\sum_{j=1}^{M}\sum_{k=1}^{M}\beta_{j}\phi_{ij}\frac{\phi_{kj}I_{k}}{N_{j}^{p}}S_{i}$$

The double summation term in this expression captures the infection at suburb *j* due to the encounters between the residents from suburb *i* and the residents from suburb *k* (*k* could be any suburb including *i* or *j*) occurring at suburb *j*, provided that suburbs i,j,k are connected with non-zero population flux entries in ϕ.

$R_{0}$ can be derived using the Next Generation Approach (see Appendix A for more details). For example, for a multi-city SEIRS model with 4 compartments (susceptible, exposed, infective, recovered), introduced in [17], and a special case where the contact rate $\lambda_{j}$ is set to 1, while β is identical across all cities, $R_{0}$ has the following analytical solution:

(11) $$R_{0}=\frac{\beta \epsilon }{\left(\gamma +d)(\epsilon +d\right)}$$

where 1/d,1/ϵ, and 1/γ denote the average lifetime, exposed period (i.e., the latent period of individuals becoming infectious) and infective period respectively. It can be seen that this solution concurs with a classical SEIRS model with no mobility. When ϵ→∞, Equation (11) reduces to β/(d+γ), being a solution for a canonical SIR model. Furthermore, if the population dynamics is not considered (i.e., d=0), Equation (11) can be further reduced to β/γ, agreeing with [19].

### 2.2. Methods

#### 2.2.1. Integrated Model

Expanding on models described in Section 2.1.2 and Section 2.1.3, we bring together population mobility and vaccinating behaviours in a network setting, and propose three extensions within an integrated vaccination-focused SIR-network model:

- vaccination is available to newborns only (model (14) below )
- vaccination is available to the entire susceptible class (model (26) below)
- committed vaccine recipients are present in the population (model (36) below)

In this study, we only consider vaccinations that confer lifelong immunity, mostly related to childhood diseases such as measles, mumps, rubella and pertussis. Vaccinations against such diseases are often administered for individuals at a young age, implying that newborns (more precisely, their parents) often face the vaccination decision. These are captured by the first extension above. However, during an outbreak, adults may face the vaccination decision themselves if they were not previously vaccinated. For example, diseases which are not included in formal childhood vaccination programs, such as smallpox [30], may present vaccination decisions to the entire susceptible population. These are captured by the second extension. The third extension introduces a small fraction of susceptible population as committed vaccine recipients, i.e., those who would always choose to vaccinate regardless of the prevalence. The purpose of modelling committed vaccine recipients is to demonstrate how successful immunization education campaigns could affect vaccination dynamics.

Our models divide the population into many homogeneous groups [31], based on their residential suburbs. Within each suburb (i.e., node), residents are treated as a homogeneous population. It is assumed that the total population within each node is conserved over time. The dynamics of epidemic and vaccination at each node, are referred to as ‘local dynamics’. The aggregate epidemic dynamics of the entire network can be obtained by summing over all nodes, producing ‘global dynamics’.

We model the ‘imitation dynamics’ based on the individual’s travelling pattern and the connectivity of their node defined by Equation (6). For any node i∈V, let $x_{i}$ denote the fraction of vaccinated individuals in the susceptible class in suburb *i*. On a particular day, unvaccinated susceptible individuals $\left(1-x_{i}\right)$ commute to suburb *j* and encounter vaccinated individuals from node *k* (where *k* may be *i* itself, *j* or any other nodes) and imitate their strategy. However, this ‘imitation’ is only applicable in the case of a non-vaccinated individual imitating the strategy of a vaccinated individual (that is, deciding to vaccinate), since the opposite ‘imitation’ cannot occur. Therefore, in our model, every time a non-vaccinated person from *i* comes in contact with a vaccinated person from *k*, they imitate the ‘vaccinate’ strategy if the perceived payoff outweighs the non-vaccination strategy.

Following Equation (4) proposed in [3], the rate of change of the proportion of vaccinated individuals in *i*, that is, $x_{i}$, over time can be expressed by:

(12) $$\begin{array}{cc} \dot{x_{i}} & =\sigma \left(1-x_{i}\right)\sum_{j=1}^{M}\sum_{k=1}^{M}\phi_{ij}\rho \Delta E\phi_{kj}x_{k}\\ & =\delta \left(1-x_{i}\right)\sum_{j=1}^{M}\sum_{k=1}^{M}\phi_{ij}\left(-r_{v}+r_{nv}mI_{j}\right)\phi_{kj}x_{k}\\ & =\kappa \left(1-x_{i}\right)\sum_{j=1}^{M}\phi_{ij}\left(-1+\omega I_{j}\right)\sum_{k=1}^{M}\phi_{kj}x_{k} \end{array}$$

The players of the vaccination game are the parents, deciding whether or not to vaccinate their children using the information of the disease prevalence collected from their daily commute. For example, a susceptible individual residing at node *i* and working at node *j* uses the local disease prevalence at node *j* to decide whether to vaccinate or not. If such a susceptible individual is infected, that individual will be counted towards the local epidemic prevalence at node *i*.

We measure each health compartment as a proportion of the population. Hence, we define a ratio, $\epsilon_{l}^{p}$, as the ratio between present population $N_{l}^{p}$ and the ‘native’ population $N_{l}$ in node *l* on a particular day as:

(13) $$\epsilon_{l}^{p}=\frac{N_{l}^{p}}{N_{l}}=\frac{\sum_{q=1}^{M}\phi_{ql}N_{q}}{N_{l}}$$

Our main focus is to investigate the effects of vaccinating behaviours on the global epidemic dynamics. To do so, we vary three parameters:

- Individual’s responsiveness to changes in disease prevalence, ω
- Adjusted imitation rate, κ
- Vaccination failure rate, ζ

while κ and ω have had been considered in [3], we introduce a new parameter ζ as the vaccination failure rate, ζ∈[0,1] to consider the cases where the vaccination may not be fully effective. The imitation component (Equation (12)) is common in all model extensions. While the epidemic compartments vary depending on the specific extension, Equation (12) is used consistently to model the relative rate of change in vaccination behaviours.

#### 2.2.2. Vaccination Available to Newborns Only

Model (14) captures the scenario when vaccination opportunities are provided to newborns only:

(14) $$\begin{array}{cc} \dot{S_{i}} & =\mu \left[\zeta x_{i}+\left(1-x_{i}\right)\right]-\sum_{j=1}^{M}\sum_{k=1}^{M}\beta_{j}\phi_{ij}\frac{\phi_{kj}I_{k}}{\epsilon_{j}^{p}}S_{i}-\mu S_{i}\\ \dot{I_{i}} & =\sum_{j=1}^{M}\sum_{k=1}^{M}\beta_{j}\phi_{ij}\frac{\phi_{kj}I_{k}}{\epsilon_{j}^{p}}S_{i}-\gamma I_{i}-\mu I_{i}\\ \dot{R_{i}} & =\mu \left(1-\zeta \right)x_{i}+\gamma I_{i}-\mu R_{i}\\ \dot{x_{i}} & =\kappa \left(1-x_{i}\right)\sum_{j=1}^{M}\phi_{ij}\left(-1+\omega I_{j}\right)\sum_{k=1}^{M}\phi_{kj}x_{k} \end{array}$$

Unvaccinated newborns and newborns with unsuccessful vaccination $\mu [\zeta x_{i}+\left(1-x_{i}\right)]$ stay in the susceptible class. Successfully vaccinated newborns, on the other hand, move to the recovered class $\mu \left(1-\zeta \right)x_{i}$. Other population dynamics across health compartments follow the model (5).

Since $S+I+R=1(\dot{S}+\dot{I}+\dot{R}=0)$, model (14) can be reduced to:

(15) $$\begin{array}{cc} \dot{S_{i}} & =\mu \left[\zeta x_{i}+\left(1-x_{i}\right)\right]-\sum_{j=1}^{M}\sum_{k=1}^{M}\beta_{j}\phi_{ij}\frac{\phi_{kj}I_{k}}{\epsilon_{j}^{p}}S_{i}-\mu S_{i}\\ \dot{I_{i}} & =\sum_{j=1}^{M}\sum_{k=1}^{M}\beta_{j}\phi_{ij}\frac{\phi_{kj}I_{k}}{\epsilon_{j}^{p}}S_{i}-\gamma I_{i}-\mu I_{i}\\ \dot{x_{i}} & =\kappa \left(1-x_{i}\right)\sum_{j=1}^{M}\phi_{ij}\left(-1+\omega I_{j}\right)\sum_{k=1}^{M}\phi_{kj}x_{k} \end{array}$$

Model (15) has a disease-free equilibrium (disease-free initial condition) $\left(S_{i}^{0},I_{i}^{0}\right)$ for which

(16) $$\begin{array}{cc} S_{i}^{0} & =\zeta x_{i}^{0}+\left(1-x_{i}^{0}\right)\\ I_{i}^{0} & =0 \end{array}$$

We can now obtain $R_{0}$ for the global dynamics in this model by using the Next Generation Approach, where $R_{0}$ is given by the most dominant eigenvalue (or ‘spectral radius’ ρ) of $FV^{-1}$, where F and V are M×M matrices, representing the ‘new infections’ and ‘cases removed or transferred from the infected class’, respectively in the disease free condition [22,32]. As a result, $R_{0}$ is determined as follows (see Appendix A for detailed derivation):

(17) $$R_{0}=\rho \left({\mathrm{FV}}^{-1}\right)$$

where

(18) $$F=\left[\begin{array}{cccc} \frac{\partial F_{1}}{\partial I_{1}} & \frac{\partial F_{1}}{\partial I_{2}} & \cdots & \frac{\partial F_{1}}{\partial I_{M}}\\ \frac{\partial F_{2}}{\partial I_{1}} & \frac{\partial F_{2}}{\partial I_{3}} & \cdots & \frac{\partial F_{2}}{\partial I_{M}}\\ \vdots & \vdots & \ddots & \vdots \\ \frac{\partial F_{M}}{\partial I_{1}} & \frac{\partial F_{M}}{\partial I_{2}} & \cdots & \frac{\partial F_{M}}{\partial I_{M}} \end{array}\right]$$

(19) $$V=\left[\begin{array}{cccc} \frac{\partial V_{1}}{\partial I_{1}} & \frac{\partial V_{1}}{\partial I_{2}} & \cdots & \frac{\partial V_{1}}{\partial I_{M}}\\ \frac{\partial V_{2}}{\partial I_{1}} & \frac{\partial V_{2}}{\partial I_{2}} & \cdots & \frac{\partial V_{2}}{\partial I_{M}}\\ \vdots & \vdots & \ddots & \vdots \\ \frac{\partial V_{M}}{\partial I_{1}} & \frac{\partial V_{M}}{\partial I_{2}} & \cdots & \frac{\partial V_{M}}{\partial I_{M}} \end{array}\right]$$

while

(20) $$\begin{array}{cc} F_{i} & =S_{i}^{0}\sum_{M}^{j=1}\sum_{M}^{k=1}\beta_{j}\phi_{ij}\frac{\phi_{kj}I_{k}^{0}}{\epsilon_{j}^{p}}\\ V_{i} & =\gamma I_{i}^{0}+\mu I_{i}^{0} \end{array}$$

Clearly, the solution of $R_{0}$ depends on $S_{i}^{0}$, $I_{k}^{0}$, epidemiological parameters (i.e., β,γ,μ), and mobility matrix ϕ. Here, we obtain an analytical expression of $R_{0}$ by studying a special case where all nodes are uniform at disease free equilibrium.

**Proposition** **1.** *At the disease free equilibrium, $x^{0}$ has two solutions ($x^{0}=0$ or $x^{0}=1$) if $x^{0}$ and $S^{0}$ are uniform across all nodes (see proof in Appendix B).*

Using Proposition 1, F can be simplified as:

(21) $$F=\beta S^{0}\left[\begin{array}{cccc} \sum_{j=1}^{M}\frac{\phi_{1j}\phi_{1j}}{\epsilon_{j}^{p}} & \sum_{j=1}^{M}\frac{\phi_{1j}\phi_{2j}}{\epsilon_{j}^{p}} & \cdots & \sum_{j=1}^{M}\frac{\phi_{1j}\phi_{Mj}}{\epsilon_{j}^{p}}\\ \sum_{j=1}^{M}\frac{\phi_{2j}\phi_{1j}}{\epsilon_{j}^{p}} & \sum_{j=1}^{M}\frac{\phi_{2j}\phi_{2j}}{\epsilon_{j}^{p}} & \cdots & \sum_{j=1}^{M}\frac{\phi_{2j}\phi_{Mj}}{\epsilon_{j}^{p}}\\ \vdots & \vdots & \ddots & \vdots \\ \sum_{j=1}^{M}\frac{\phi_{Mj}\phi_{1j}}{\epsilon_{j}^{p}} & \sum_{j=1}^{M}\frac{\phi_{Mj}\phi_{2j}}{\epsilon_{j}^{p}} & \cdots & \sum_{j=1}^{M}\frac{\phi_{Mj}\phi_{Mj}}{\epsilon_{j}^{p}} \end{array}\right]$$

We can now prove the following simple but useful proposition (see proof in Appendix B).

**Proposition** **2.** *Matrix G, defined as follows:*

(22) $$G=\left[\begin{array}{cccc} \sum_{j=1}^{M}\frac{\phi_{1j}\phi_{1j}}{\epsilon_{j}^{p}} & \sum_{j=1}^{M}\frac{\phi_{1j}\phi_{2j}}{\epsilon_{j}^{p}} & \cdots & \sum_{j=1}^{M}\frac{\phi_{1j}\phi_{Mj}}{\epsilon_{j}^{p}}\\ \sum_{j=1}^{M}\frac{\phi_{2j}\phi_{1j}}{\epsilon_{j}^{p}} & \sum_{j=1}^{M}\frac{\phi_{2j}\phi_{2j}}{\epsilon_{j}^{p}} & \cdots & \sum_{j=1}^{M}\frac{\phi_{2j}\phi_{Mj}}{\epsilon_{j}^{p}}\\ \vdots & \vdots & \ddots & \vdots \\ \sum_{j=1}^{M}\frac{\phi_{Mj}\phi_{1j}}{N_{j}^{p^{*}}} & \sum_{j=1}^{M}\frac{\phi_{Mj}\phi_{2j}}{N_{j}^{p^{*}}} & \cdots & \sum_{j=1}^{M}\frac{\phi_{Mj}\phi_{Mj}}{N_{j}^{p^{*}}} \end{array}\right]$$

*is a Markov matrix.*

As a Markov matrix, G always has the most dominant eigenvalue of unity. All other eigenvalues are smaller than unity in absolute value [33].

Now the next generation matrix K can be obtained as follows:

(23) $$\begin{array}{cc} K & =\frac{\beta S^{0}}{\gamma +\mu }G\\ & =\frac{\beta [\left(1-x^{0}\right)+\zeta x^{0}]}{\gamma +\mu }G \end{array}$$

From Proposition 2 and Equation (17), $R_{0}$ can be obtained as:

(24) $$\begin{array}{cc} R_{0} & =\rho (K)\\ & =\frac{\beta [\left(1-x^{0}\right)+\zeta x^{0}]}{\gamma +\mu }\rho \left(G\right)\\ & =\frac{\beta [\left(1-x^{0}\right)+\zeta x^{0}]}{\gamma +\mu } \end{array}$$

Noting Proposition 1, there are two cases: x=0 and x=1. When nobody vaccinates ($x^{0}=0,$$S^{0}=1$), the entire population remains susceptible, which reduces model (15) to a canonical SIR model without vaccination intervention and $R_{0}$ returns to $\frac{\beta }{\gamma +\mu }$. On the other hand, if the whole population is vaccinated ($x^{0}=1$), the fraction of susceptible population only depends on the vaccine failure rate ζ, resulting in:

(25) $$R_{0}=\frac{\beta \zeta }{\gamma +\mu }$$

Clearly, fully effective vaccination (ζ=0) would prohibit disease spread ($R_{0}=0$). Partially effective vaccination could potentially suppress disease transmission, or even eradicate disease spread if $R_{0}<1$. If all vaccinations fail (ζ=1), model (15) concurs with a canonical SIR model without vaccination, which also corresponds to a special case in Equation (11) where ϵ→∞.

#### 2.2.3. Vaccination Available to the Entire Susceptible Class

If the vaccination opportunity is expanded to the entire susceptible class, including newborns and adults, the following model is proposed based on model (15):

(26) $$\begin{array}{cc} \dot{S_{i}} & =\mu -\sum_{j=1}^{M}\sum_{k=1}^{M}\beta_{j}\phi_{ij}\frac{\phi_{kj}I_{k}}{\epsilon_{j}^{p}}S_{i}-\mu S_{i}-x_{i}S_{i}+x_{i}\zeta S_{i}\\ \dot{I_{i}} & =\sum_{j=1}^{M}\sum_{k=1}^{M}\beta_{j}\phi_{ij}\frac{\phi_{kj}I_{k}}{\epsilon_{j}^{p}}S_{i}-\gamma I_{i}-\mu I_{i}\\ \dot{R_{i}} & =S_{i}x_{i}\left(1-\zeta \right)+\gamma I_{i}-\mu I_{i}\\ \dot{x_{i}} & =\kappa \left(1-x_{i}\right)\sum_{j=1}^{M}\phi_{ij}\left(-1+\omega I_{j}\right)\sum_{k=1}^{M}\phi_{kj}x_{k} \end{array}$$

Model (26) has a disease-free equilibrium $\left(S_{i}^{0},I_{i}^{0},x_{i}^{0}\right)$:

(27) $$\begin{array}{cc} S_{i}^{0} & =\frac{\mu }{\mu +x_{i}^{0}\left(1-\zeta \right)}\\ I_{i}^{0} & =0 \end{array}$$

while

(28) $$\begin{array}{cc} F_{i} & =S_{i}^{0}\sum_{j=1}^{M}\sum_{k=1}^{M}\beta_{j}\phi_{ij}\frac{\phi_{kj}I_{k}^{0}}{\epsilon_{j}^{p}}\\ V_{i} & =\gamma I_{i}^{0}+\mu I_{i}^{0} \end{array}$$

Substituting $S_{i}^{0}$ using Equation (16) yields

(29) $$\begin{array}{cc} F & =\beta \frac{\mu }{\mu +x^{0}\left(1-\zeta \right)}G\\ V & =(\gamma +\mu )I \end{array}$$

where I is a M×M identity matrix.

The next generation matrix K can then be obtained as:

(30) $$K=\frac{\beta \mu }{\left(\gamma +\mu \right)[\mu +x^{0}\left(1-\zeta \right)]}G$$

Using Proposition 2, $R_{0}$ can be obtained as:

(31) $$R_{0}=\frac{\beta \mu }{\left(\gamma +\mu \right)[\mu +x^{0}\left(1-\zeta \right)]}$$

When nobody vaccinates ($x^{0}=0$), model (26) concurs with a canonical SIR model with $R_{0}$ reducing to $\frac{\beta }{\gamma +\mu }$. When the vaccination attains the full coverage in the population ($x^{0}=1$), the magnitude of $R_{0}$ depends on the vaccine failure rate ζ:

(32) $$R_{0}=\frac{\beta \mu }{\left(\gamma +\mu )(\mu +1-\zeta \right)}$$

Equation (32) reduces to $\frac{\beta }{\gamma +\mu }$ if all vaccines fail (ζ=1). Conversely, if all vaccines are effective ζ=0, Equation (32) yields

(33) $$R_{0}=\frac{\beta \mu }{\left(\gamma +\mu )(\mu +1\right)}$$

Given that μ≪1, $R_{0}$ is well below the critical threshold:

(34) $$R_{0}=\frac{\beta \mu }{\gamma }\ll 1$$

If 0<ζ<1, by comparing Equations (25) and (32), we note that $R_{0}$ becomes smaller, provided ζ>μ, that is:

(35) $$\begin{array}{cc} R_{0}^{newborn}-R_{0}^{susceptible} & =\frac{\beta \zeta }{\gamma +\mu }-\frac{\beta \mu }{\left(\gamma +\mu )(\mu +1-\zeta \right)}\\ & =\frac{\beta (1-\zeta )(\zeta -\mu )}{\left(\gamma +\mu )(\mu +1-\zeta \right)}>0 \end{array}$$

#### 2.2.4. Vaccination Available to the Entire Susceptible Class with Committed Vaccine Recipients

We now consider the existence of committed vaccine recipients, $x^{c}$, as a fraction of individuals who would choose to vaccinate regardless of payoff assessment [34,35] ($0<x^{c}\ll 1$). We assume that committed vaccine recipients are also exposed to vaccination failure rate ζ and are distributed uniformly across all nodes. It is also important to point out that the fraction of committed vaccine recipients is constant over time. However, they can still affect vaccination decision for those who are not vaccinated, and consequently, contribute to the rate of change of the vaccinated fraction *x*. Model (26) can be further extended to reflect these considerations, as follows:

(36) $$\begin{array}{cc} \dot{S_{i}} & =\mu -\sum_{j=1}^{M}\sum_{k=1}^{M}\beta_{j}\phi_{ij}\frac{\phi_{kj}I_{k}}{\epsilon_{j}^{p}}S_{i}-\mu S_{i}+\left(\zeta -1\right)\left[\left(x_{i}+x^{c}\right)S_{i}\right]\\ \dot{I_{i}} & =\sum_{j=1}^{M}\sum_{k=1}^{M}\beta_{j}\phi_{ij}\frac{\phi_{kj}I_{k}}{\epsilon_{j}^{p}}S_{i}-\gamma I_{i}-\mu I_{i}\\ \dot{x_{i}} & =\kappa \left(1-x_{i}-x^{c}\right)\sum_{j=1}^{M}\phi_{ij}\left(-1+\omega I_{j}\right)\sum_{k=1}^{M}\phi_{kj}\left(x_{k}+x^{c}\right) \end{array}$$

Model (36) has a disease-free equilibrium:

(37) $$\begin{array}{cc} S_{i}^{0} & =\frac{\mu }{\mu +\left(x_{i}^{0}+x^{c}\right)\left(1-\zeta \right)}\\ I_{i}^{0} & =0 \end{array}$$

while

(38) $$\begin{array}{cc} F_{i} & =S_{i}^{0}\sum_{M}^{j=1}\sum_{M}^{k=1}\beta_{j}\phi_{ij}\frac{\phi_{kj}I_{k}^{0}}{\epsilon_{j}^{p}}\\ V_{i} & =\gamma I_{i}^{0}+\mu I_{i}^{0} \end{array}$$

Substituting $S_{i}^{0}$, using Equation (37), yields:

(39) $$\begin{array}{cc} F & =\frac{\beta \mu }{\mu +\left(x^{0}+x^{c}\right)\left(1-\zeta \right)}\left[\begin{array}{cccc} \sum_{j=1}^{M}\frac{\phi_{1j}\phi_{1j}}{\epsilon_{j}^{p}} & \sum_{j=1}^{M}\frac{\phi_{1j}\phi_{2j}}{\epsilon_{j}^{p}} & \cdots & \sum_{j=1}^{M}\frac{\phi_{1j}\phi_{Mj}}{\epsilon_{j}^{p}}\\ \sum_{j=1}^{M}\frac{\phi_{2j}\phi_{1j}}{\epsilon_{j}^{p}} & \sum_{j=1}^{M}\frac{\phi_{2j}\phi_{2j}}{\epsilon_{j}^{p}} & \cdots & \sum_{j=1}^{M}\frac{\phi_{2j}\phi_{Mj}}{\epsilon_{j}^{p}}\\ \vdots & \vdots & \ddots & \vdots \\ \sum_{j=1}^{M}\frac{\phi_{Mj}\phi_{1j}}{\epsilon_{j}^{p}} & \sum_{j=1}^{M}\frac{\phi_{Mj}\phi_{2j}}{\epsilon_{j}^{p}} & \cdots & \sum_{j=1}^{M}\frac{\phi_{Mj}\phi_{Mj}}{\epsilon_{j}^{p}} \end{array}\right]\\ V & =\left(\gamma +\mu \right)I \end{array}$$

where I is a M×M identity matrix.

The next generation matrix K can now be obtained as:

(40) $$K=\frac{\beta \mu }{\left[\mu +\left(x^{0}+x^{c}\right)\left(1-\zeta \right)\right]\left(\gamma +\mu \right)}G$$

**Proposition** **3.** *At the disease free equilibrium, $x^{0}$ has two solutions $x^{0}=x^{c}=0$ or $x^{0}+x^{c}=1$ if $x^{0}$, $x^{c}$ and $S^{0}$ are uniform across all nodes.*

$R_{0}$ can be obtained for this case as:

(41) $$R_{0}=\frac{\beta \mu }{\left[\mu +\left(x^{0}+x^{c}\right)\left(1-\zeta \right)\right]\left(\gamma +\mu \right)}$$

If nobody chooses to vaccinate ($x^{0}=x^{c}=0$), $R_{0}$ can be reduced to $\frac{\beta }{\gamma +\mu }$. Conversely, if the entire population is vaccinated $\left(x^{0}+x_{c}=1\right)$, Equation (41) reduces to Equation (32), and $R_{0}$ is purely dependent on the vaccine failure rate ζ.

#### 2.2.5. Model Parameterisation

The proposed models aim to simulate a scenario of a generic childhood disease (e.g., measles) where life-long full immunity is acquired after effective vaccination. The vaccination failure rate, ζ, is set as the probability of ineffective vaccination, to showcase the influence of unsuccessful vaccination on the global epidemic dynamics. In reality, the vaccination for Measles-Mumps-Rubella (MMR) is highly effective: for example, in Australia, an estimated 96% of vaccines that were administered are successful in conferring immunity [36].

The population flux matrix ϕ is derived from the network topology, in which each entry represents the connectivity between two nodes: if two nodes are not connected, $\phi_{ij}=0$; if two nodes are connected, $\phi_{ij}$ is randomly assigned in the range of (0,1]. The population influx into a node *i* within a day is represented by the column sum $\sum_{j=1}^{M}\phi_{ji}$ in flux matrix ϕ. The entries of ϕ are determined by network topologies.

The parameters used for all simulations are summarized in Table 1. Same initial conditions are applied to all nodes. Parameters ω and κ are calibrated based on values used in [3]. Here, we aim to investigate how vaccination behaviours affect overall epidemic dynamics. To do so, we mainly study the effects of two parameters on all models: ω, responsiveness to changes in disease prevalence; and κ, imitation rate (i.e., how quickly individuals imitate each other).

Our simulations were carried out on three networks:

- a pilot case of a network with 3 nodes (suburbs),
- an Erdös-Rényi random network [38] with 3000 nodes (suburbs), and
- the commuting network in Greater Sydney with 311 suburbs (nodes) [39,40].

Each of the first two networks was used in conjunction with the following three models of vaccination behaviours:

- vaccinating newborns only: using model (15)
- vaccinating the susceptible class: using model (26),
- vaccinating the susceptible class with committed vaccine recipients: using model (36).

The third network is a real-world network and is considered a more realistic representation of travelling patterns. It was only used in conjunction with model (15) where only newborns are vaccinated as the existing immunisation programmes in Australia typically administer measles vaccines to newborns.

**Figure 2:**
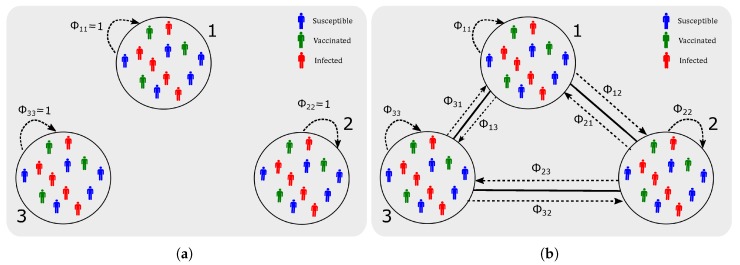
Schematic of the 3-node case: population mobility across nodes. (**a**) No population mobility. $i=j=\left\{1,2,3\right\},\phi_{ij}=0$ where i≠j. Otherwise $\phi_{ij}=1$. (**b**) Equal population mobility. $i=j=\left\{1,2,3\right\},\phi_{ij}=\frac{1}{3}$.

In the pilot case of 3-node network, two network topologies are studied: an isolated 3-node network where all residents remain in their residential nodes without travelling to other nodes (equivalent to models proposed in [3]), and a fully connected network where the residents at each node commute to the other two nodes, and the population fractions commuting are symmetric and uniformly distributed (Figure 2).

We then consider a suburb-network modelled as an Erdös-Rényi random graph (with number of nodes M=3000) with average degree 〈k〉=4, in order to study how an expansive travelling pattern affects the global epidemic dynamics. (See Figure 10 for the degree distribution of the network used.) Other topologies, such as lattice, scale-free [41,42], small-world networks and other real-world networks [20,43], can easily be substituted here, though in this study our focus is on an Erdös-Rényi random graphs.

## 3. Results

### 3.1. 3-Node Network

#### 3.1.1. Vaccinating Newborns Only

**Figure 3:**
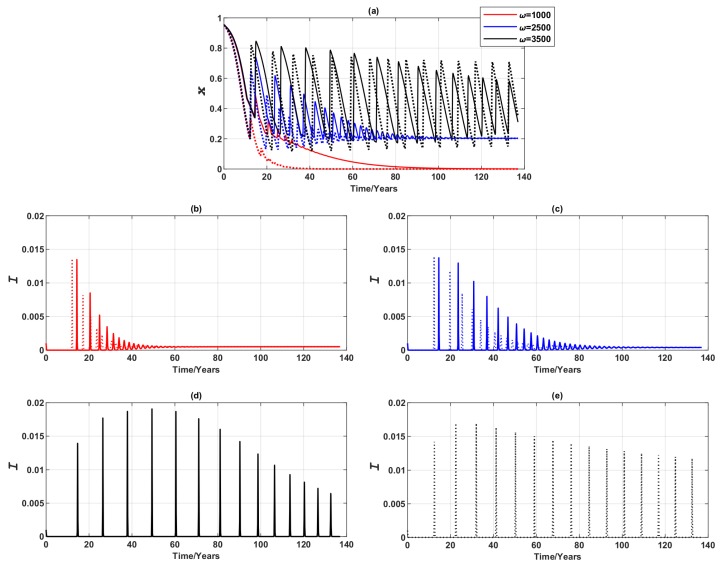
Epidemic dynamics of a 3-node case for three values of ω when vaccinating newborns. Time series of (**a**) the relative proportion of vaccinated individuals, *x*, and (**b**–**e**) Infection prevalence, *I*. Solid line: Symmetric uniform population mobility. Dotted line: No population mobility. Commuting suppresses prevalence peaks over time at high ω, but may produce higher prevalence peaks over time at mid and low ω.

When there is no population mobility, we observe three distinctive equilibria (Figure 3; dotted lines): a pure non-vaccinating equilibrium (where $x^{f}=0$, representing the final condition, and ω=1000), a mixed equilibrium (where $x^{f}\neq 0$, ω=2500), and stable limit cycles (where $x^{f}\neq 0$, ω=3500). This observation is in qualitative agreement with the vaccinating dynamics reported by [3].

When commuting is allowed, individuals commute to different nodes, and their decision will no longer rely on the single source of information (i.e., the disease prevalence in their residential node) but will also depend on the disease prevalence at their destination. As a consequence, the three distinctive equilibria are affected in different ways (Figure 3; solid lines). The amplitude of the stable limit cycles at ω=3500 is reduced as a result of the reduced disease prevalence. It takes comparatively longer (compare the dotted lines and solid lines in Figure 3) to converge to the pure non-vaccinating equilibrium at ω=1000, and to the endemic, mixed equilibrium at ω=2500 with high amplitude of oscillation at the start of the epidemic spread. As ω can be interpreted as the responsiveness of vaccinating behaviour to the disease prevalence [3], if individuals are sufficiently responsive (i.e., ω is high), the overall epidemic is suppressed more due to population mobility (and the ensuing imitation), as evidenced by smaller prevalence peaks. Conversely, if individuals are insensitive to the prevalence change (i.e., ω is low), epidemic dynamics with equal population mobility may appear to be more volatile at the start, but the converged levels of both prevalence peak and vaccine uptake remain unchanged in comparison to the case where there is no population mobility.

**Figure 4:**
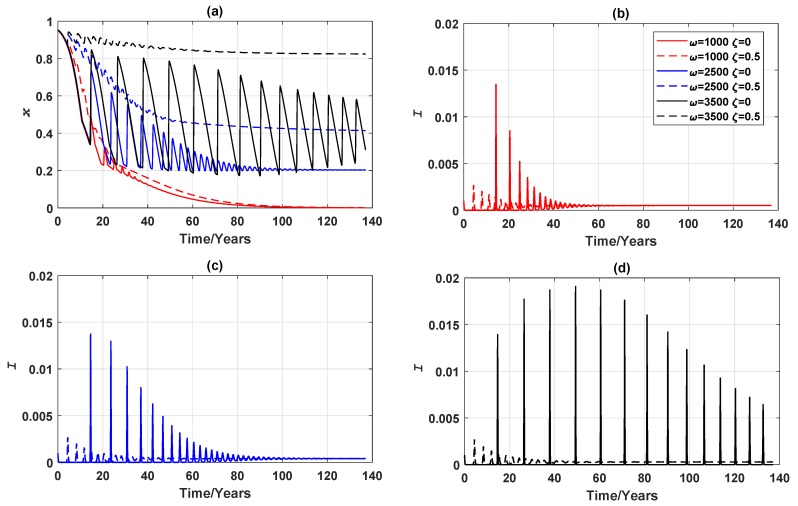
Comparison of vaccination failure rates: epidemic dynamics of a 3-node case for three values of ω (which measures the responsiveness of individuals to prevalence) when vaccinating newborns. (**a**) Relative proportion of vaccinated individuals, *x*, and (**b**–**d**) Disease prevalence (i.e., Proportion of infected individuals), *I*. Solid line: ζ=0. Dashed line: ζ=0.5.

We further investigated how the vaccination failure rate ζ affects the global epidemic dynamics. If, for example, only a half of the vaccine administered is effective (ζ=0.5), as shown in Figure 4b–d, the infection peaks arrive sooner, for all values of ω. As a result of having the earlier infection peaks, the individuals respond to the breakout and choose to vaccinate sooner, causing the vaccine uptake to rise. This seemingly counter-intuitive behaviours have also been reported in [44]. When ζ=0, the prevalence peaks take longer to develop and the extended period gives individuals an illusion that there may not be an epidemic breakout, and consequently encourages ‘free-riding’ behaviour. In the case where half of the vaccinations fail, epidemic breaks out significantly earlier at lower peaks, an observation that is beneficial to encourage responsive individuals to choose to vaccinate. Although some (in this case half) of the vaccines fail, a sufficiently high vaccine uptake still curbs prevalence peaks and shortens the length of breakout period. In terms of the final vaccination uptake, the vaccine failure rate predominantly affects behaviours of responsive individuals (when ω is high, such as 3500). In this case, instead of the stable limit cycles observed when ζ=0, an endemic, mixed equilibrium is reached when the vaccination failure rate is significant. Final vaccination uptake is impacted little by vaccination failure rate when individuals are insensitive to changes in disease prevalence (when ω is lower in value, such as 2500 or 1000). These observations are illustrated in Figure 4.

#### 3.1.2. Vaccinating the Entire Susceptible Class

**Figure 5:**
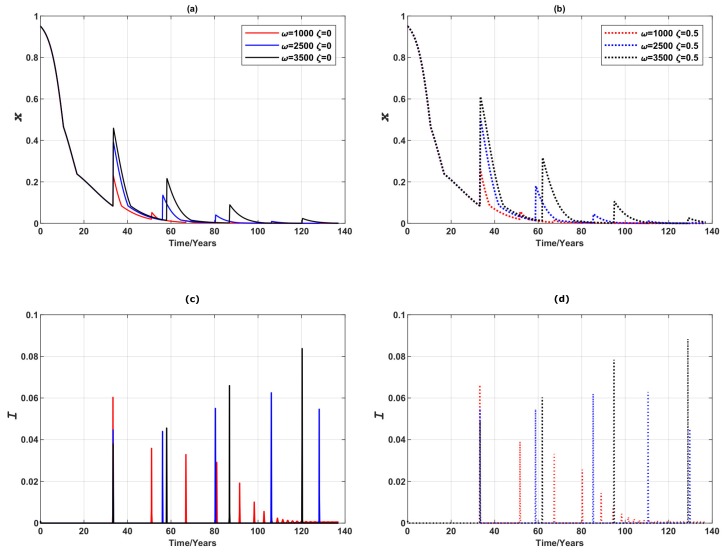
Comparison of vaccination failure rates: epidemic dynamics of a 3-node case for three values of ω (which measures the responsiveness of individuals to prevalence) when vaccinating newborns and adults. (**a**,**b**) Relative proportion of vaccinated individuals, *x*, and (**c**,**d**) Disease prevalence (i.e., proportion of infected individuals), *I*. Solid line: ζ=0. Dashed line: ζ=0.5.

If (voluntary) vaccination is offered to the susceptible class regardless of the age, the initial condition of x=0.95 represents the scenario that the vast majority of population are immunized to begin with, and the epidemic would not breakout until the false sense of security provided by the temporary ‘herd immunity’ settles in. This feature is observed in Figure 5a,b: the vaccination coverage continues to drop at the start of the epidemic breakout, indicating that individuals, regardless of their responsiveness towards the prevalence change, exploit the temporary herd immunity until an infection peak emerges. Since vaccination is available to the entire susceptible class, a small increase in x could help suppresses disease prevalence. However, when vaccination is partially effective (i.e., ζ=0.5), the peaks in vaccine uptake, x, are no longer an accurate reflection of the actual vaccination coverage, and such peaks therefore may not be sufficient to adequately suppress infection peaks. It can also be observed that vaccinating the susceptible class encourages non-vaccinating behaviours due to the perception of herd immunity, and therefore pushes the epidemic towards an endemic equilibrium, particularly when sensitivity to prevalence is relatively high ( mid and high ω), as the responsive individuals would react promptly to the level of disease prevalence, altering their vaccination decisions. However, no substantial impact is observed on those individuals who are insensitive to changes in the disease prevalence ( low ω).

### 3.2. Erdös-Rényi Random Network of 3000 Nodes

We now present the simulation results on a much larger Erdö-Rényi random network of M=3000 nodes, which more realistically reflects the size of a modern city and its commuting patterns. It was observed by previous studies [45] that such a larger system requires a higher vaccination coverage to achieve herd immunity, and thus curbs ‘free-riding’ behaviours more effectively.

#### 3.2.1. Vaccinating Newborns Only

**Figure 6:**
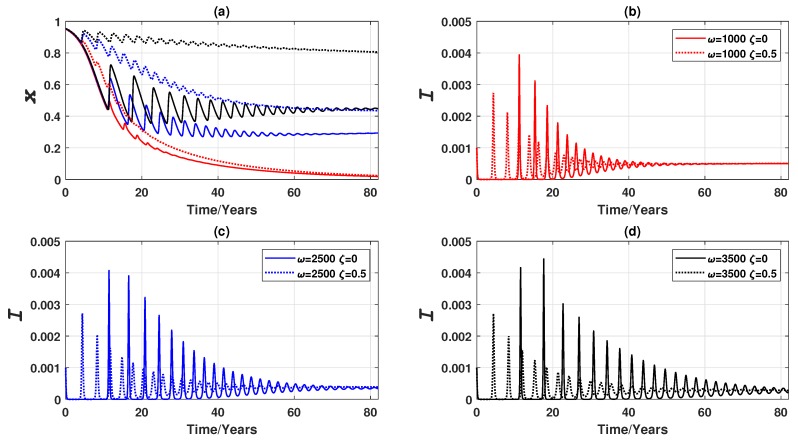
Comparison of vaccination failure rates: epidemic dynamics of a Erdös-Rényi random network of 3000 nodes for three values of ω (which measures the responsiveness of individuals to prevalence) when vaccinating newborns. (**a**) Relative proportion of vaccinated individuals, *x*, and (**b**–**d**) Disease prevalence (i.e., proportion of infected individuals), *I*. Solid line: ζ=0. Dashed line: ζ=0.5.

In this case, three distinct equilibria are observed (as shown in Figure 6a) for three values of ω: a pure non-vaccinating equilibrium at ω=1000 and two endemic mixed equilibria at ω=2500 and ω=3500 respectively, replacing the stable limit cycles at high ω previously observed in the 3-node case. As expected, when individuals are more responsive to disease prevalence (higher ω), they are more likely to get vaccinated. The expansive travelling pattern also somewhat elevates the global vaccination coverage level (compared to the 3-node case), particularly in the case that the individuals show a moderate level of responsiveness to prevalence (ω=2500), and shortens the convergence time to reach the equilibrium. However, for those who are insensitive to the changes in disease prevalence (ω=1000), the more expansive commuting presented by the larger network does not affect either the level of voluntary vaccination, or the convergence time in global dynamics—these individuals remain unvaccinated as in the case of the smaller network. It is also found that the vaccination coverage is very sensitive to the disease prevalence change at the larger network since the disease prevalence peaks are notably lower than the 3-node case counterparts. If half of the vaccines administered are unsuccessful (ζ=0.5), the impact of early peaks on global dynamics is magnified in a larger network (Figure 6b–d), leading to a shorter convergence time, although the final equilibria are hardly affected compared to the 3-node case as shown in Figure 4.

**Figure 7:**
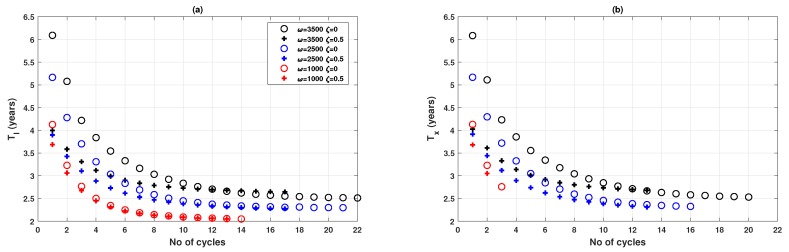
Oscillation properties of epidemic and vaccination dynamics for three values of ω when vaccinating newborns. (**a**) Period of the disease prevalence, $T_{I}$, and (**b**) Period of the relative proportion of vaccinated individuals, $T_{x}$. Circle: ζ=0; plus sign: ζ=0.5. A peak, for the purpose of measuring period, is defined by a peak threshold θ: $\theta_{I}=0.0001$ for *I*, and $\theta_{x}=0.01$ for *x*.

It is observed that the shape of oscillation, in terms of amplitude and period, varies with different values of ω. Figure 6a (also Figure 4 for a much smaller network) show that lower ω is related with higher damping ratio in the oscillatory dynamics of the disease prevalence *I* and the vaccination coverage *x*. A closer inspection reveals that the oscillations observed are non-harmonic with the period reducing after every cycle (Figure 7a,b). Also, period is positively correlated with the value of ω, an observation supported by some preliminary analysis as reported in Appendix C, indicating that the time interval between prevalence peaks is longer if population is sufficiently responsive to prevalence change. Such a conclusion is not affected by the vaccine failure rate ζ, although the period is noticeably smaller at the start of the outbreak when ζ is larger (ζ=0.5) (Figure 7c,d). Furthermore, it is worth noting that the oscillatory properties may also be affected by epidemiological parameters, such as transmission rate β, a shown in Appendix C.

**Figure 8:**
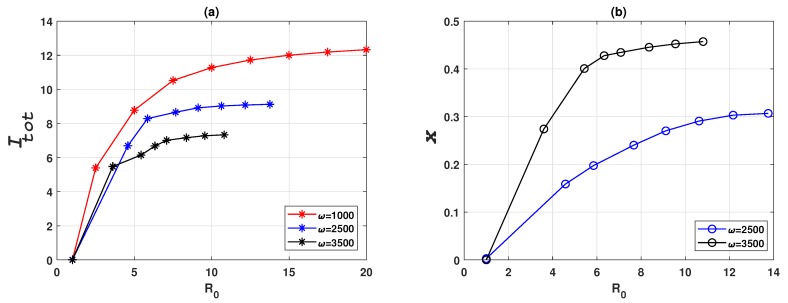
Epidemic and vaccination dynamics of a Erdös-Rényi random network of 3000 nodes for various values of basic reproduction number $R_{0}$, for three values of ω. (**a**) Cumulative prevalence, $I_{tot}$. (**b**) Relative proportion of vaccinated individuals, *x*. $R_{0}$ is varied by varying the infection rate β. Cumulative prevalence $I_{tot}$ is obtained by integrating the prevalence over the simulated time frame. Note that different ω settings correspond to different ranges for $R_{0}$ due to the different vaccination coverage, *x*, at their respective endemic equilibria. Note that in (**b**) the case for ω=1000 is not shown because it is trivially zero for all values of $R_{0}$.

To verify the analytical derivation of $R_{0}$, we investigated the relationship between epidemic and vaccination dynamics and the basic reproduction ratio $R_{0}$. According to Equation (24), higher vaccination coverage leads to a reduction of $R_{0}$. Figure 8 confirms this analytical dependency by showing that higher vaccination coverage (higher *x*), reduces $R_{0}$ and consequently leads to smaller cumulative prevalence, provided individuals are sufficiently responsive towards prevalence change (i.e., ω=2500 and ω=3500). When population is insensitive to prevalence change (ω=1000), the vaccination dynamics always converge to the non-vaccination equilibrium, corresponding to the case where x=0 in Equation (24).

**Figure 9:**
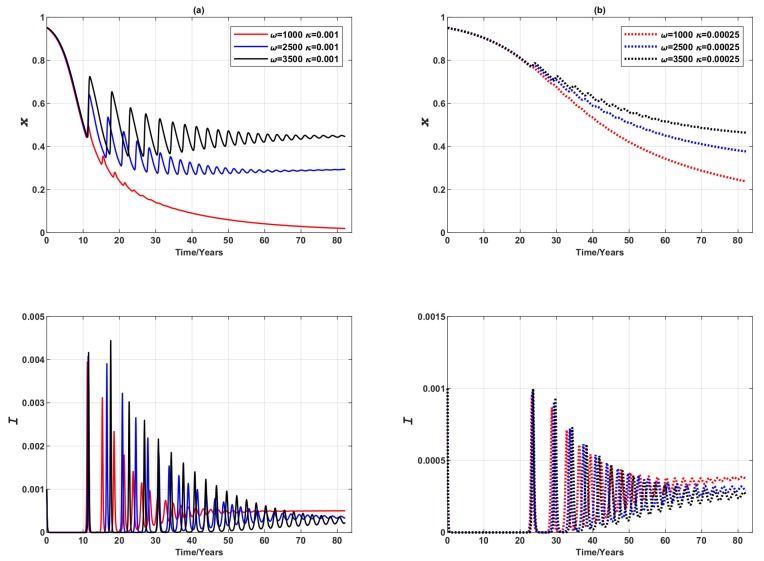
Epidemic dynamics of a Erdös-Rényi random network of 3000 nodes, varying the value of κ for three values of ω (vaccinating newborns). Time series of relative proportion of vaccinated individuals, *x*, and disease prevalence (i.e., proportion of infected individuals), *I*. (**a**) Solid line: κ=0.001. (**b**) Dotted line: κ=0.00025.

We also compared the epidemic dynamics in terms of the adjusted imitation rate, represented by the parameter κ, as shown in Figure 9 (recall that the value of imitation rate used in our simulations, unless otherwise stated, is κ=0.001 as reported in Table 1). Here we present results where this value of imitation rate is compared with a much smaller value of κ=0.00025. In populations where the imitation rate is comparatively high (i.e., higher κ), the oscillations in prevalence and vaccination dynamics appear earlier in time with larger amplitude, although the converged vaccine uptake and disease prevalence are relatively unaffected by the value of κ. For a higher κ, the convergence to equilibria is quicker. These observations are consistent with results reported by earlier studies [3]. A smaller value of κ (κ=0.00025) also altered behaviours of those individuals who are insensitive to prevalence change. Instead of converging to the pure non-vaccination equilibrium, the dynamics converge to a mixed endemic state, meaning that when imitation rate is very low, some individuals would still choose to vaccinate even when they are not very sensitive to disease prevalence.

**Figure 10:**
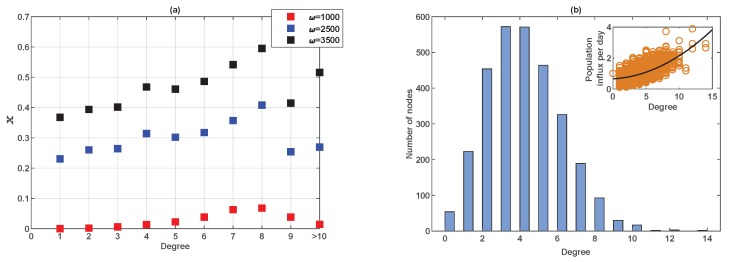
The relationship between node degree and proportion of people who vaccinate voluntarily (vaccinating newborns only) for an Erdös-Rényi random network of 3000 nodes. (**a**) The fraction of vaccinated individuals as a function of node degree (which is the number of neighbouring suburbs for each suburb considered) has for three values of ω. (**b**) The degree distribution of the Erdös-Rényi random network. The inset figure shows the population influx per node (sum of flux fractions from each source node) as a function of the node degree.

For such a large network, vaccinating behaviour of individuals also depends on the weighted degree (number and weight of connections) of the suburb (node) in which they live. This is illustrated in Figure 10. For individuals living in highly connected suburbs (nodes), there are many commuting destinations, allowing access to a broad spectrum of information on local disease prevalence. Therefore, it is not surprising that we found that individuals living in ‘hubs’ are more likely to get vaccinated, particularly when the population is sensitive to prevalence (ω is high), as shown in Figure 10a. This observation is in accordance with previous studies [6]. Note that in in Figure 10a, nodes with degree k≥10 are grouped into one bin to represent hubs, as the frequency count of these nodes is extremely low. Note also that there is a positive correlation between the number of degrees and the volume of population influx as measured by the sum of proportions from the source nodes (Figure 10b), indicating that a highly connected suburb has greater population influx on a daily basis.

**Figure 11:**
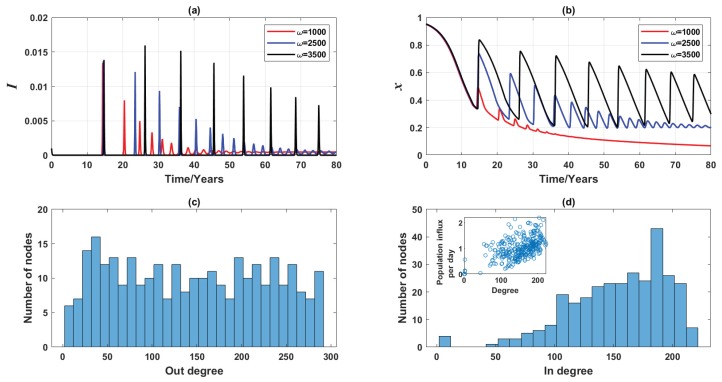
Simulated dynamics (vaccinating newborns only) of the commuting network in Greater Sydney generated from the 2016 Australian census data, for three values of ω. Time series of (**a**) disease prevalence, *I*, (**b**) relative proportion of vaccinated individuals, *x*, (**c**) out-degree distribution of the network (representing population outflux), and (**d**) in-degree distribution of the network (representing population influx). The inset figure shows the population influx per node as a function of the node degree. Other network properties: M=311,〈k〉≈150.

To verify that the above mentioned simulation experiments reasonably model real-world scenarios, we performed additional experiments using a real-world commuting network of Greater Sydney generated from the 2016 Australian census data [39,40,46,47]. In contrast with agent-based models [46,47], in which commuting individuals interact at their workplaces or schools only with other commuters, rather than with the residential population, we consider a general case where commuting and residential populations are well-mixed at every node. This assumption provides a generic insight into how empirical networks affect epidemic and vaccination dynamics. As illustrated in Figure 11d, the real commuting network is substantially denser (M=311,〈k〉≈150) in comparison with the studied Erdös-Rényi random network. While the three distinctive equilibria still hold, it takes longer for insensitive individuals (i.e., ω=1000) to reach non-vaccinating equilibrium (Figure 11b), an observation that is not previously noted in the 3-node pilot case and 3000-node Erdös-Rényi random network. In general, however, the dynamics produced on this real-world network concur with the results obtained for previously described simulation experiments.

#### 3.2.2. Vaccinating Entire Susceptible Class

**Figure 12:**
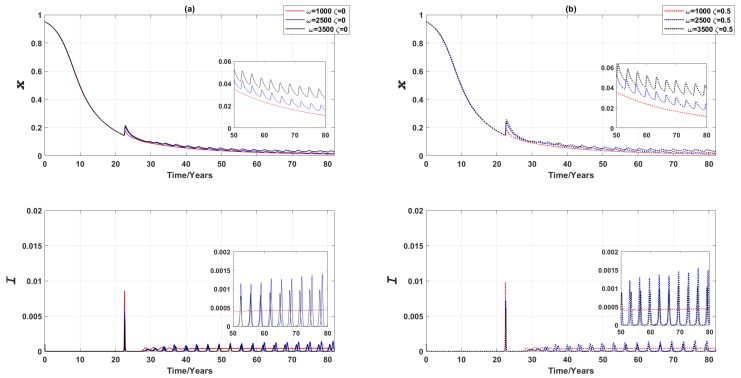
Epidemic dynamics of a Erdös-Rényi random network of 3000 nodes for three values of ω (vaccinating susceptible class regardless of age). Relative proportion of vaccinated individuals, *x*, and disease prevalence (i.e., the proportion of infected individuals), *I*, are shown against time. (**a**) ζ=0 (**b**) ζ=0.5. The inset figure in each figure is a magnified section to show small oscillations.

If (voluntary) vaccination is offered to the susceptible class regardless of age, we found that the global epidemic dynamics converge quicker compared to the similar scenario in the 3-node case. Only one predominant infection peak is observed, corresponding to the high infection prevalence around year 25, as shown in Figure 12. Oscillations of small magnitude are observed for both vaccine uptake and disease prevalence at later time-steps for middle or high values of ω (as shown in insets of Figure 12). These findings also largely hold if half of the vaccines administered are ineffective (i.e., ζ=0.5), although the predominant prevalence peak and the corresponding vaccination peak around year 25 are both higher than their counterparts observed for the case where ζ=0.

**Figure 13:**
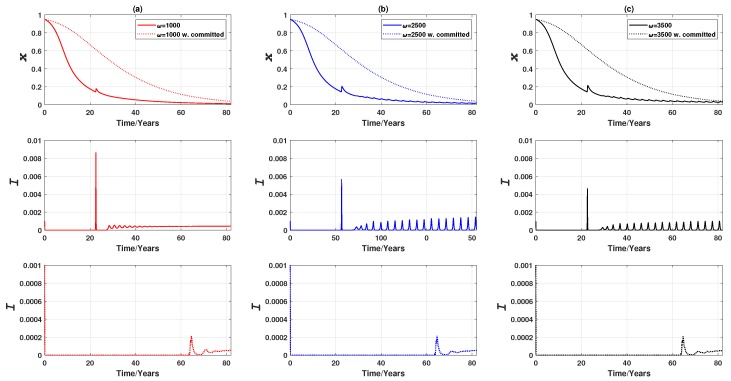
Epidemic dynamics of a Erdös-Rényi random network of 3000 nodes with committed vaccine recipients for three values of ω (vaccinating the entire susceptible class). Relative proportion of vaccinated individuals, *x*, against time, and disease prevalence (i.e., the proportion of infected individuals), *I*, against time. (**a**) ω=1000 (**b**) ω=2500 (**c**) ω=3500. Solid line: without committed vaccine recipients. Dotted line: with committed vaccine recipients. The proportion of committed vaccine recipients, $x^{c}=0.0002$. The existence of committed vaccine recipients delays the predominant peaks and reduces the magnitude of oscillation in the proportion of vaccine recipients in later stages.

Furthermore, We found that employing a small fraction of committed vaccine recipients prevents a major epidemic by curbing disease prevalence. Such a finding holds for all ω (i.e., regardless of the population’s responsiveness towards disease prevalence) as the magnitude of prevalence is too small to make a non-vaccinated individual switch to vaccinating (Figure 13).

## 4. Discussion and Conclusions

We presented a series of SIR-network models with imitation dynamics, aiming to model scenarios where individuals commute between their residence and work, which is modelled by a commuting network where each node represents a suburb. These network models are able to capture diverse travelling patterns (i.e., reflecting local connectivity of suburbs), and different vaccinating behaviours affecting the global vaccination uptake and epidemic dynamics. We also analytically derived expressions for the basic reproduction number $R_{0}$ for the considered SIR-network models, and demonstrated how epidemics may evolve over time in these models.

We showed that the stable oscillations in the vaccinating dynamics are only likely to occur either when there is no population mobility across nodes, or only with limited commuting destinations. We observed that, compared to the case where vaccination is only provided to newborns, if vaccination is provided to the entire susceptible class, higher disease prevalence and more volatile oscillations in vaccination uptake are observed (particularly in populations which are relatively responsive to the changes in disease prevalence). A more expansive travelling pattern simulated in a larger network leads to the appearance of attractor dynamics in the relative proportion of vaccinated individuals, *x*, and the proportion of infected individuals, *I*, and the eventual convergence to the endemic, mixed equilibria, again if individuals are sufficiently responsive towards the changes in the disease prevalence. If individuals are insensitive to the prevalence, they are hardly affected by different vaccinating models and remain as unvaccinated individuals, although the existence of committed vaccine recipients noticeably delays the convergence to the non-vaccinating equilibrium. The presented models highlight the important role of committed vaccine recipients in actively reducing $R_{0}$ and disease prevalence, strongly contributing to eradicating an epidemic spread. Similar conclusions have been reached previously [34], and our results extend these to imitation dynamics in SIR-network models.

Previous studies drew an important conclusion that highly connected hubs play a key role in containing infections as they are more likely to get vaccinated due to the higher risk of infection in social networks [6,45]. Our results, verified by simulation experiments on a Greater Sydney commuting network, complement this finding by showing that a higher fraction of individuals who reside in highly connected suburbs (nodes) choose to vaccinate compared to those living in relatively less connected suburbs (nodes). These hubs, often recognized as business districts, also have significantly higher population influx as the destination for many commuters from other suburbs. Therefore, it is important for policy makers to leverage these job hubs in promoting vaccination campaigns and public health programs.

Overall, our results demonstrate that, in order to encourage vaccination behaviour and shorten the course of epidemic, policy makers need to carefully balance the following three considerations: ensuring a number of committed vaccine recipients exist in each suburb, utilizing the fact that people well-connected suburbs are more likely to vaccinate, and increasing individual awareness towards the prevalence change.

There are several avenues to extend this work further. This work assumes that the individuals from different suburbs (nodes) only differ in their travelling patterns, using the same epidemic and behaviour parameters for individuals from all nodes. Also, the same ω and κ are used for all nodes, by assuming that individuals living in all suburbs (nodes) are equally responsive towards disease prevalence and imitation. A greater level of accuracy in modelling can be achieved by establishing context-specific $R_{0}$ and imitating parameters for factors such as the local population density, community size [37], travelling rate [21], and suburbs’ level of connectivity. For example, residents living in highly connected suburbs may be more alert to changes in disease prevalence, and adopt imitation behaviours more quickly. Bounded rationality can also be used to consider cases where individuals are not perfectly rational [48,49]. Different network topologies can also be used, particularly scale-free networks [41,42] where a small number of nodes have a large number of links each. These highly connected nodes are a better representation of suburbs with extremely high population influx (e.g., central business districts and job hubs). Dependency between epidemic dynamics and network topologies/properties are also of strong interest. Further investigation can be conducted by calibrating the network to real-world networks (other than the Greater Sydney commuting network that we studied here) to mimic a breakout in a targeted geographical region, such as a city, a suburb, or a statistical local area [46,47,50]. Previous studies also revealed that network topological metrics such as centrality measures, assortativity and robustness play an important role in the epidemic dynamics [51,52,53,54]. Further refinement on network-based modelling can also be attempted by introducing multiplex networks [55] where epidemic spreading and social interactions rely on two separate networks. It may also be instructive to translate the risk perception of vaccination and infection into tangible measures to demonstrate the aggregate social cost of an epidemic breakout, and help policy makers to visualize the cost effectiveness of different vaccinating strategies and estimate the financial burden for public health care. Parameters can also be calibrated to model other diseases with rich epidemic data, such as 2009 H1N1 influenza pandemic and 2003 SARS epidemic [43]. These considerations could advance this line of research to more accurately reflect contagion dynamics in urban environments, and provide further insights to public health planning.

## Figures and Tables

**Table 1:** Epidemiological and behavioural parameters.

Parameter	Interpretation	Baseline Value	References
1/γ	Average length of recovery period (days)	10	[37]
$R_{0}$	Basic reproduction number	15	[37]
μ	Mean birth and death rate (days${}^{-1}$)	0.000055	[3]
ζ	Vaccination failure rate	[0,1]	Assumed
κ	Imitation rate	0.001	[3]
ω	Responsiveness to changes in disease prevalence	[1000,3500]	[3]
$\phi_{ij}$	Fraction of residents from node *i* travelling to *j*	[0,1]	Network connectivity
			(See Figures 10b and 11d
			for degree distribution.)
*I*	Initial condition	0.001	[3]
*S*	Initial condition	0.05	[3]
*x*	Initial condition	0.95	[3]

**Figure A1:**
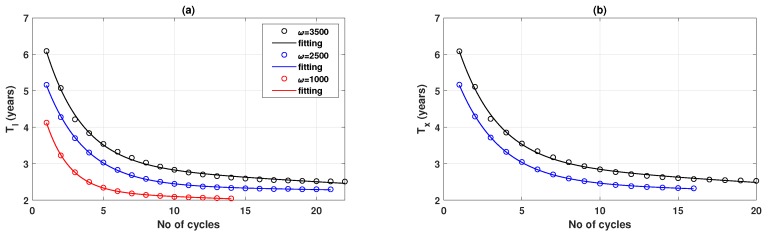
The function $f\left(x\right)=ae^{bx}+ce^{dx}$ is fitted for the period of epidemic and vaccination dynamics for three values of ω. (**a**) Period of the disease prevalence, $T_{I}$. (**b**) Period of the relative proportion of vaccinated individuals, $T_{x}$. Circle: original data point. Solid line: fitted curve. Results of goodness-of-fit test are summarised in Table A1. Coefficients of fitting functions are summarised in Table A2.

**Figure A2:**
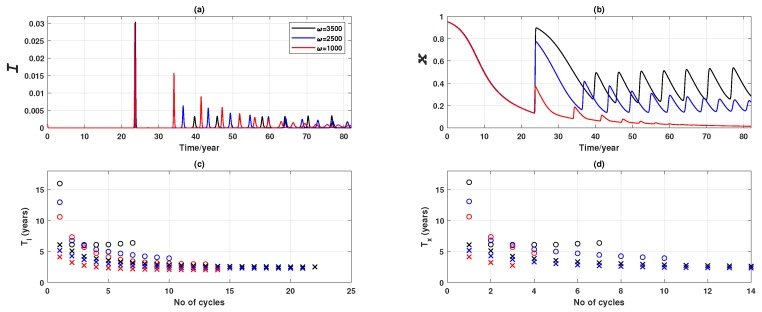
Oscillation properties of epidemic and vaccination dynamics for three values of ω when vaccinating newborns at low β (β=0.75, or $R_{0}=7.5$). Note that for the results reported in the main body of the paper, the transmission rate β was 1.5. (**a**) Disease prevalence (i.e., proportion of infected individuals), *I*, (**b**) Relative proportion of vaccinated individuals, *x*, (**c**) comparison of the period of the disease prevalence, $T_{I}$, at different β, and (**d**) comparison of period of the relative proportion of vaccinated individuals, $T_{x}$, at different β. Circle: β=0.75. Cross: β=1.5. A peak, for the purpose of measuring period, is defined by a peak threshold θ: $\theta_{I}=0.0001$ for *I*, and $\theta_{x}=0.01$ for *x*.

## Appendix A. Next Generation Operator Approach

The proposed model (15) can be seen as a finely categorized SIR deterministic model, so that each health compartment (Susceptible, Infected and Recovered) has *M* sub-classes. Let us denote $I=\{I_{1},I_{2},\ldots ,I_{M}\}$ to represent *M* infected host compartments, and y to represent 2M other host compartments consisting of susceptible compartments $y_{s}=\left\{y_{s1},y_{s2},\ldots ,y_{sM}\right\}$ and recovered compartments $y_{r}=\left\{y_{r1},y_{r2},\ldots ,y_{rM}\right\}$.

(A1) $$\begin{array}{cc} \frac{dI_{i}}{dt} & =F_{i}\left(I,y_{s}\right)-V_{i}\left(I,y_{r}\right)\\ \frac{dy_{s}}{dt} & =G_{sj}\left(I,y_{s}\right)\\ \frac{dy_{r}}{dt} & =G_{rj}\left(I,y_{r}\right) \end{array}$$

where i∈(1,M) and j∈(1,M).

$F_{i}$ is the rate at which new infection enters infected compartments and $V_{i}$ is the transfer of individuals out of or into the infected compartments.

When close to disease-free equilibrium where $S=S^{*}=1$, the model can be linearized to:

(A2) $$\frac{dI}{dt}=\left(F-V\right)I$$

where $F_{ij}=\frac{\partial F_{i}}{\partial I_{j}}\left(0,S^{*}\right)$ and $V_{ij}=\frac{\partial V_{i}}{\partial I_{j}}\left(0,S^{*}\right)$

The next generation matrix, K, is then given by:

(A3) $$K={\mathrm{FV}}^{-1}$$

where each entry $K_{ij}$ represents the expected number of secondary cases which an infected individual imposes on the rest of the compartments. F and V are given as follows:

(A4) $$F=\left[\begin{array}{cccc} \frac{\partial F_{1}}{\partial I_{1}} & \frac{\partial F_{1}}{\partial I_{2}} & \cdots & \frac{\partial F_{1}}{\partial I_{M}}\\ \frac{\partial F_{2}}{\partial I_{1}} & \frac{\partial F_{2}}{\partial I_{2}} & \cdots & \frac{\partial F_{2}}{\partial I_{M}}\\ \vdots & \vdots & \ddots & \vdots \\ \frac{\partial F_{M}}{\partial I_{1}} & \frac{\partial F_{M}}{\partial I_{2}} & \cdots & \frac{\partial F_{M}}{\partial I_{M}} \end{array}\right]$$

(A5) $$V=\left[\begin{array}{cccc} \frac{\partial V_{1}}{\partial I_{1}} & \frac{\partial V_{1}}{\partial I_{2}} & \cdots & \frac{\partial V_{1}}{\partial I_{M}}\\ \frac{\partial V_{2}}{\partial I_{1}} & \frac{\partial V_{2}}{\partial I_{2}} & \cdots & \frac{\partial V_{2}}{\partial I_{M}}\\ \vdots & \vdots & \ddots & \vdots \\ \frac{\partial V_{M}}{\partial I_{1}} & \frac{\partial V_{M}}{\partial I_{2}} & \cdots & \frac{\partial V_{M}}{\partial I_{M}} \end{array}\right]$$

The basic reproduction number $R_{0}$ is given by the most dominant eigenvalue (or ‘spectral radius’ ρ) of K [22,31,32], and therefore:

(A6) $$R_{0}=\rho \left(K\right)$$

## Appendix B. Propositions and Proofs

**Proposition** **A1.**
*At the disease free equilibrium, $x^{0}$ has two solutions ($x^{0}=0$ or $x^{0}=1$) if $x^{0}$ and $S^{0}$ are uniform across all nodes.*

**Proof.** At the disease free equilibrium, assuming $S_{i}^{0},x_{i}^{0}$ are uniform across all nodes, and therefore:

(A7) $$S_{i}^{0}=S^{0},\;x_{i}^{0}=x^{0}\;\forall i$$

Hence, under disease-free condition where $I^{0}=0$ and $\dot{x_{i}}=0$, Equation (15) becomes:

(A8) $$\begin{array}{c} 0=\left(1-x_{i}^{0}\right)x_{i}^{0}\sum_{j=1}^{M}\phi_{ij}\left(-1+\omega I_{j}\right)\sum_{k=1}^{M}\phi_{kj} \end{array}$$

Equation (A8) leads to two solutions: $x^{0}=0$ or $x^{0}=1$. □

Assuming $\beta_{i}=\beta$ for all nodes, we can derive F and V as follows:

(A9) $$\begin{array}{cc} F & =\beta \left[\begin{array}{cccc} \sum_{j=1}^{M}S_{1}^{0}\frac{\phi_{1j}\phi_{1j}}{\epsilon_{j}^{p}} & \sum_{j=1}^{M}S_{1}^{0}\frac{\phi_{1j}\phi_{2j}}{\epsilon_{j}^{p}} & \cdots & \sum_{j=1}^{M}S_{1}^{0}\frac{\phi_{1j}\phi_{Mj}}{\epsilon_{j}^{p}}\\ \sum_{j=1}^{M}S_{2}^{0}\frac{\phi_{2j}\phi_{1j}}{\epsilon_{j}^{p}} & \sum_{j=1}^{M}S_{2}^{0}\frac{\phi_{2j}\phi_{2j}}{\epsilon_{j}^{p}} & \cdots & \sum_{j=1}^{M}S_{2}^{0}\frac{\phi_{2j}\phi_{Mj}}{\epsilon_{j}^{p}}\\ \vdots & \vdots & \ddots & \vdots \\ \sum_{j=1}^{M}S_{M}^{0}\frac{\phi_{Mj}\phi_{1j}}{\epsilon_{j}^{p}} & \sum_{j=1}^{M}S_{M}^{0}\frac{\phi_{Mj}\phi_{2j}}{\epsilon_{j}^{p}} & \cdots & \sum_{j=1}^{M}S_{M}^{0}\frac{\phi_{Mj}\phi_{Mj}}{\epsilon_{j}^{p}} \end{array}\right]\\ V & =\left(\gamma +\mu \right)I \end{array}$$

where I is a M×M identity matrix.

**Proposition** **A2.**
*Matrix G, defined as follows:*

(A10) $$G=\left[\begin{array}{cccc} \sum_{j=1}^{M}\frac{\phi_{1j}\phi_{1j}}{\epsilon_{j}^{p}} & \sum_{j=1}^{M}\frac{\phi_{1j}\phi_{2j}}{\epsilon_{j}^{p}} & \cdots & \sum_{j=1}^{M}\frac{\phi_{1j}\phi_{Mj}}{\epsilon_{j}^{p}}\\ \sum_{j=1}^{M}\frac{\phi_{2j}\phi_{1j}}{\epsilon_{j}^{p}} & \sum_{j=1}^{M}\frac{\phi_{2j}\phi_{2j}}{\epsilon_{j}^{p}} & \cdots & \sum_{j=1}^{M}\frac{\phi_{2j}\phi_{Mj}}{\epsilon_{j}^{p}}\\ \vdots & \vdots & \ddots & \vdots \\ \sum_{j=1}^{M}\frac{\phi_{Mj}\phi_{1j}}{N_{j}^{p^{*}}} & \sum_{j=1}^{M}\frac{\phi_{Mj}\phi_{2j}}{N_{j}^{p^{*}}} & \cdots & \sum_{j=1}^{M}\frac{\phi_{Mj}\phi_{Mj}}{N_{j}^{p^{*}}} \end{array}\right]$$

*is a Markov matrix.*

**Proof.** We assume all nodes have the same population *N*. Then Equation (13) can be reduced to:

(A11) $$\epsilon_{i}^{p^{*}}=\sum_{k=1}^{M}\phi_{ki}$$

Without loss of generality, substituting Equation (A11) into Equation (A10) and expanding entries in the first column yields the first column $g_{1}$ of matrix G:

(A12) $$g_{1}=\left[\begin{array}{cc} \frac{\phi_{11}\phi_{11}}{\sum_{k=1}^{M}\phi_{k1}}+\frac{\phi_{12}\phi_{12}}{\sum_{k=1}^{M}\phi_{k2}}+\cdots +\frac{\phi_{1M}\phi_{1M}}{\sum_{k=1}^{M}\phi_{kM}} & \cdots \\ \frac{\phi_{21}\phi_{11}}{\sum_{k=1}^{M}\phi_{k1}}+\frac{\phi_{22}\phi_{12}}{\sum_{k=1}^{M}\phi_{k2}}+\cdots +\frac{\phi_{2M}\phi_{1M}}{\sum_{k=1}^{M}\phi_{kM}} & \cdots \\ \vdots & \vdots \\ \frac{\phi_{M1}\phi_{11}}{\sum_{k=1}^{M}\phi_{k1}}+\frac{\phi_{M2}\phi_{12}}{\sum_{k=1}^{M}\phi_{k2}}+\cdots +\frac{\phi_{MM}\phi_{1M}}{\sum_{k=1}^{M}\phi_{kM}} & \cdots \end{array}\right]$$

Continuing with column 1, the column sum is:

(A13) $$\begin{array}{c} =\phi_{11}\frac{\sum_{k=1}^{M}\phi_{k1}}{\sum_{k=1}^{M}\phi_{k1}}+\phi_{12}\frac{\sum_{k=1}^{M}\phi_{k2}}{\sum_{k=1}^{M}\phi_{k2}}+\cdots +\phi_{1M}\frac{\sum_{k=1}^{M}\phi_{kM}}{\sum_{k=1}^{M}\phi_{kM}}\\ =\sum_{k=1}^{M}\phi_{1k}=1 \end{array}$$

Similarly, the sums of each column are equal to 1, with all entries being non-negative population fractions. Hence, G is a Markov matrix. □

**Proposition** **A3.**
*At the disease free equilibrium, $x^{0}$ has two solutions $x^{0}=x^{c}=0$ or $x^{0}+x^{c}=1$ if $x^{0}$, $x^{c}$ and $S^{0}$ are uniform across all nodes.*

**Proof.** In analogy with Proposition A1, with committed vaccine recipients, at the disease free equilibrium, $S_{i}^{0}$ and $x_{i}^{0}$ are uniform across all nodes, and therefore:

(A14) $$S_{i}^{0}=S^{0},\;x_{i}^{0}=x^{0}+x^{c}\;\forall i$$

Hence, under disease-free condition where $I^{0}=0$ and $\dot{x_{i}}=0$, Equation (36) becomes:

(A15) $$\begin{array}{cc} 0 & =\left(1-x_{i}^{0}-x^{c}\right)\left(x_{i}^{0}+x^{c}\right)\sum_{j=1}^{M}\phi_{ij}\left(-1+\omega I_{j}\right)\sum_{k=1}^{M}\phi_{kj} \end{array}$$

Equation (A15) leads to two solutions: $x^{0}+x^{c}=0$ or $x^{0}+x^{c}=1$. □

## Appendix C. Preliminary Analysis of the Oscillatory Behaviour of Epidemic and Vaccination Dynamics When Vaccinating Newborns

We found that a two-term exponential function ($f\left(x\right)=ae^{bx}+ce^{dx}$) is a satisfactory fit for Figure 7 as shown in Figure A1 and Table A1. Note that we do not fit a function for the case of ω=1000 because of insufficient data points.

Table A2 provides some preliminary insight on how ω affects the length of period. The length of period is relatively unchanged for epidemic and vaccination dynamics despite different oscillatory profiles. ω is positively correlated to the period, indicating that the time interval between prevalence peaks is longer if population is sufficiently responsive to prevalence change.

**Table A1:** Goodness-of-fit test of curves shown in Figure A1. Results rounded to 4 significant figures. SSE: sum of squared errors of prediction. RMSE: Root Mean Square Error.

$T_{I}$	**SSE**	**R-Square**	**Adjusted R-Square**	**RMSE**
ω=3500	0.03564	0.9980	0.9977	0.04450
ω=2500	0.002712	0.9998	0.9997	0.01260
ω=1000	0.0008870	0.9980	0.9997	0.009418
$T_{x}$	**SSE**	**R-Square**	**Adjusted R-Square**	**RMSE**
ω=3500	0.03076	0.9982	0.9979	0.04385
ω=2500	0.001248	0.9999	0.9998	0.01020

**Table A2:** Coefficients of two-term exponential fitted functions. Results are with 95% confidence bounds, shown in bracket.

$T_{I}$	**a**	**b**	**c**	**d**
ω=3500	4.593 (4.406,4.78)	−0.3882 (−0.4247,−0.3516)	2.983 (2.832,3.134)	−0.008774 (−0.01176,−0.005788)
ω=2500	3.951 (3.9,4.003)	−0.3616 (−0.3732,−0.35)	2.399 (2.353,2.446)	−0.00239 (−0.003547,−0.001234)
ω=1000	3.468 (3.398,3.538)	−0.6031 (−0.628,−0.5782)	2.237 (2.195,2.28)	−0.006735 (−0.008434,−0.005035)
$T_{x}$	**a**	**b**	**c**	**d**
ω=3500	4.535 (4.341,4.73)	−0.3971 (−0.4385,−0.3557)	3.066 (2.889,3.244)	−0.01052 (−0.0142,−0.006853)
ω=2500	3.882 (3.827,3.937)	−0.3717 (−0.3857,−0.3577)	2.492 (2.422,2.561)	−0.004952 (−0.006928,−0.002976)

We undertook an additional simulation experiment with a low transmission rate (β=0.75), corresponding to a case where the disease is less infectious. Figure A2a,b show that the mixed, endemic equilibria at mid and high ω (ω=2500 and ω=3500) observed in Figure 6, in this case, are replaced by stable limit cycles, while the convergence to the pure, non-vaccination equilibrium at low ω (ω=1000) is relatively unaffected. The reduced β also delays the first prominent peak with noticeably longer period between two peaks, as shown in Figure A2. Despite different oscillatory profiles, we note that higher ω generally corresponds to longer period, indicating that more responsive individuals extend the period between two epidemic peaks due to the higher vaccination coverage.

## References

[1] World Heath Organization (2006). Basic Epidemiology.

[2] Jansen V.A.A., Stollenwerk N., Jensen H., Ramsay M., Edmunds W., Rhodes C. (2003). Measles outbreaks in a population with declining vaccine uptake. Science.

[3] Bauch C.T. (2005). Imitation dynamics predict vaccinating behaviour. Proc. R. Soc. B.

[4] Wang Z., Bauch C., Bhattacharyya S., d’Onofrio A., Manfredi P., Perc M.P.N., Salathè M., Zhao D. (2016). Statistical physics of vaccination. Phys. Rep..

[5] Bauch C.T., Bhattacharyya S. (2012). Evolutionary game theory and social learning can determine how vaccine scares unfold. PLoS Comput. Biol..

[6] Fu F., Rosenbloom D., Wang L., Nowak M. (2010). Imitation dynamics of vaccination behaviour on social networks. Proc. R. Soc. B.

[7] Liu X., Wu Z., Zhang L. (2012). Impact of committed individuals on vaccination behaviour. Phys. Rev. E.

[8] Zhang H., Fu F., Zhang W., Wang B. (2012). Rational behaviour is a ‘double-edged sword’ when considering voluntary vaccination. Physica A.

[9] Zhang Y. (2013). The impact of other-regarding tendencies on the spatial vaccination network. Chaos Solitons Fractals.

[10] Li Q., Li M., Lv L., Guo C., Lu K. (2017). A new prediction model of infectious diseases with vaccination strategies based on evolutionary game theory. Chaos Solitons Fractals.

[11] Feng X., Bin W., Long W. (2018). Voluntary vaccination dilemma with evolving psychological perceptions. J. Theor. Biol..

[12] Bauch C., Earn D.J.D. (2004). Vaccination and the theory of games. Proc. Natl. Acad. Sci. USA.

[13] Bauch C., Earn D.J.D. (2003). Transients and attractors in epidemics. Proc. R. Soc. B.

[14] Bauch C., Galvani A.P., Earn D. (2003). Group interest versus self-interest in smallpox vaccination policy. Proc. Natl. Acad. Sci. USA.

[15] Perisic A., Bauch C. (2009). A simulation analysis to characterize the dynamics of vaccinating behaviour on contact networks. BioMed Cent..

[16] Wang Z., Andrews M., Wu Z., Wang L., Bauch C. (2015). Coupled disease–behavior dynamics on complex networks: A review. Phys. Life Rev..

[17] Arino J., van den Driessche P., Benvenuti L., de Santis A., Farina L. (2003). The Basic Reproduction Number in a Multi-city Compartmental Epidemic Model.

[18] Arino J., van den Driessche P. (2003). A multi-city epidemic model. Math. Popul. Stud..

[19] Stolerman L., Coombs D., Boatto S. (2015). SIR-network model and its application to dengue fever. J. Appl. Math..

[20] Pei S., Kandula S., Yang W., Shaman J. (2018). Forecasting the spatial transmission of influenza in the United States. Proc. Natl. Acad. Sci. USA.

[21] Belik V., Geisel T., Brockmann D. (2011). Natural Human Mobility Patterns and Spatial Spread of Infectious Diseases. Phys. Rev. X.

[22] Diekmann O., Heesterbeek J.A.P., Metz J.A.J. (1990). On the definition and the computation of the basic reproduction ratio *R*_0_ in models for infectious diseases in heterogeneous populations. J. Math. Biol..

[23] Anderson R.M., May R.M. (1991). Infectious Diseases of Humans.

[24] Harding N., Nigmatullin R., Prokopenko M. (2018). Thermodynamic efficiency of contagions: A statistical mechanical analysis of the SIS epidemic model. Interface Focus.

[25] Hartfield M., Alizon S. (2013). Introducing the Outbreak Threshold in Epidemiology. PLoS Pathog..

[26] Pastor-Satorras R., Castellano C., Mieghem P., Vespignani A. (2015). Epidemic processes in complex networks. Rev. Mod. Phys..

[27] Van den Driessche P., Watmough J., Wu J. (2008). Further notes on the basic reproduction number. Mathematical Epidemiology.

[28] Erten E., Lizier J., Piraveenan M., Prokopenko M. (2017). Criticality and information dynamics in epidemiological models. Entropy.

[29] Brauer F., Wu J. (2008). Compartmental Models in Epidemiology. Mathematical Epidemiology.

[30] Australian Government, Department of Health (2018). National Immunisation Program Schedule. https://beta.health.gov.au/health-topics/immunisation/immunisation-throughout-life/national-immunisation-program-schedule.

[31] Van den Driessche P., Watmough J. (2002). Reproduction numbers and sub-threshold endemic equilibria for compartmental models of disease transmission. Math. Biosci..

[32] Diekmann O., Heesterbeek J., Roberts M.G. (2010). The construction of next-generation matrices for compartmental epidemic models. J. R. Soc. Interface.

[33] Seneta E. (1981). Non-Negative Matrices and Markov Chains.

[34] Bai Z. (2015). Global dynamics of a SEIR model with information dependent vaccination and periodically varying transmission rate. Math. Methods Appl. Sci..

[35] Buonomo B., d’Onofrio A., Lacitignola D. (2013). Modelling of pseudo-rational exemption to vaccination for SEIR diseases. J. Math. Anal. Appl..

[36] Sheppeard V., Forssman B., Ferson M., Moreira C., Campbell-Lloyd S., Dwyer D., McAnulty J. (2009). Vaccine failures and vaccine effectiveness in children during measles outbreaks in New South Wales, March–May 2006. Commun. Dis. Intell. (CDI).

[37] Guerra F., Bolotin S., Lim G., Heffernan J., Deeks S., Li Y., Crowcroft N. (2017). The basic reproduction number (*R*_0_) of measles: A systematic review. Lancet Infect. Dis..

[38] Erdös P., Rényi A. (1959). On random graphs. Publ. Math..

[39] Australian Bureau of Statistics (2017). Census of Population and Housing: Understanding the Census and Census Data, Australia, 2016.

[40] Fair K., Zachreson C., Prokopenko M. (2019). Creating a surrogate commuter network from Australian Bureau of Statistics census data. Sci. Data.

[41] Cardillo A., Reyes-Suárez C., Naranjo F., Gómez-Gardeñes J. (2013). Evolutionary vaccination dilemma in complex networks. Phys. Rev. E.

[42] Barabási A. (2016). Scale-free Properties. Network Science.

[43] Brockmann D., Helbing D. (2013). The Hidden Geometry of Complex, Network-Driven Contagion Phenomena. Science.

[44] Steinegger B., Cardillo A., Rios P., Gómez-Gardeñes J., Arenas A. (2018). Interplay between cost and benefits triggers nontrivial vaccination uptake. Phys. Rev. E.

[45] Pastor-Satorras R., Vespignani A. (2002). Immunization of complex networks. Phys. Rev. E.

[46] Cliff O.M., Harding N., Piraveenan M., Erten Y., Gambhir M., Prokopenko M. (2018). Investigating spatiotemporal dynamics and synchrony of influenza epidemics in Australia: An agent-based modelling approach. Simul. Model. Pract. Theory.

[47] Zachreson C., Fair K.M., Cliff O.M., Harding N., Piraveenan M., Prokopenko M. (2018). Urbanization affects peak timing, prevalence, and bimodality of influenza pandemics in Australia: Results of a census-calibrated model. Sci. Adv..

[48] Kasthurirathna D., Piraveenan M. (2015). Emergence of scale-free characteristics in socio-ecological systems with bounded rationality. Sci. Rep..

[49] Kasthurirathna D., Piraveenan M., Uddin S. (2016). Modeling networked systems using the topologically distributed bounded rationality framework. Complexity.

[50] Balcan D., Vespignani D. (2011). Phase transitions in contagion processes mediated by recurrent mobility patterns. Nat. Phys..

[51] Piraveenan M., Prokopenko M., Hossain L. (2013). Percolation Centrality: Quantifying Graph-Theoretic Impact of Nodes during Percolation in Networks. PLoS ONE.

[52] Thedchanamoorthy G., Piraveenan M., Uddin S., Senanayake U. (2014). Influence of vaccination strategies and topology on the herd immunity of complex networks. Soc. Netw. Anal. Min..

[53] Piraveenan M., Prokopenko M., Zomaya A. (2009). Assortativeness and information in scale-free networks. Eur. Phys. J. B.

[54] Badham J., Stocker R. (2010). The impact of network clustering and assortativity on epidemic behaviour. Theor. Popul. Biol..

[55] Granell C., Gómez S., Arenas A. (2013). Dynamical Interplay between Awareness and Epidemic Spreading in Multiplex Networks. Phys. Rev. Lett..

